# Coenzyme Q10 eyedrops conjugated with vitamin E TPGS alleviate neurodegeneration and mitochondrial dysfunction in the diabetic mouse retina

**DOI:** 10.3389/fncel.2024.1404987

**Published:** 2024-05-28

**Authors:** Christie Hang-I Lam, Bing Zuo, Henry Ho-Lung Chan, Tsz-Wing Leung, Samuel Abokyi, Kirk Patrick Carreon Catral, Dennis Yan-Yin Tse

**Affiliations:** ^1^School of Optometry, The Hong Kong Polytechnic University, Kowloon, Hong Kong SAR, China; ^2^Centre for Eye and Vision Research Limited, Shatin, Hong Kong SAR, China; ^3^Research Centre for SHARP Vision, The Hong Kong Polytechnic University, Kowloon, Hong Kong SAR, China

**Keywords:** diabetic retinopathy, mitochondrial dysfunction, neurodegeneration, Coenzyme Q10, electroretinography

## Abstract

Diabetic retinopathy (DR) is a leading cause of blindness and vision impairment worldwide and represents one of the most common complications among diabetic patients. Current treatment modalities for DR, including laser photocoagulation, intravitreal injection of corticosteroid, and anti-vascular endothelial growth factor (VEGF) agents, target primarily vascular lesions. However, these approaches are invasive and have several limitations, such as potential loss of visual function, retinal scars and cataract formation, and increased risk of ocular hypertension, vitreous hemorrhage, retinal detachment, and intraocular inflammation. Recent studies have suggested mitochondrial dysfunction as a pivotal factor leading to both the vascular and neural damage in DR. Given that Coenzyme Q10 (CoQ10) is a proven mitochondrial stabilizer with antioxidative properties, this study investigated the effect of CoQ10 eyedrops [in conjunction with vitamin E d-α-tocopheryl poly(ethylene glycol) 1000 succinate (TPGS)] on DR-induced neurodegeneration using a type 2 diabetes mouse model (C57BLKsJ-db/db mice). Utilizing a comprehensive electroretinography protocol, supported by immunohistochemistry, our results revealed that topical application of CoQ10 eyedrops conjugated with vitamin E TPGS produced a neuroprotective effect against diabetic-induced neurodegeneration by preserving the function and histology of various retinal neural cell types. Compared to the control group, mice treated with CoQ10 exhibited thicker outer and inner nuclear layers, higher densities of photoreceptor, cone cell, and rod-bipolar cell dendritic boutons, and reduced glial reactivity and microglial cell density. Additionally, the CoQ10 treatment significantly alleviated retinal levels of MMP-9 and enhanced mitochondrial function. These findings provide further insight into the role of mitochondrial dysfunction in the development of DR and suggest CoQ10 eyedrops, conjugated with vitamin E TPGS, as a potential complementary therapy for DR-related neuropathy.

## 1 Introduction

Diabetic retinopathy (DR) is one of the most common complications of diabetic patients (Simó-Servat et al., 2019). The diagnosis and severity grading of DR depends ultimately on the presence of vascular lesions, including, but not limited to, retinal hemorrhages, exudates, venous beading/reduplication, and intraretinal microvascular abnormalities. Indeed, a growing body of evidence suggests that diabetes affects the entire neurovascular unit of the eye and diabetes-induced neurodegeneration may occur prior to the overt retinal vascular abnormalities and could contribute to the development of the vasculopathy (Barber, 2003; Antonetti et al., 2006; Abcouwer and Gardner, 2014).

Currently, the mainstay medical treatment of DR is intravitreal injection of anti-vascular endothelial growth factor (VEGF) agents. This approach has been found to cause less tissue damage and complications than laser photocoagulation and intravitreal injection of corticosteroid (Gupta et al., 2013). However, this treatment modality has several limitations. Firstly, anti-VEGF agents have been shown to cause neurotrophic factor deprivation and adversely affect retinal neural cells in streptozotocin-induced diabetic rodents (Park et al., 2014). Secondly, the efficacy of anti-VEGF agents varies among individuals (Heier et al., 2016). Thirdly, the invasive procedure of intravitreal injection needs to be repeated at regular intervals, which discourages compliance. Lastly, it is associated with increased risks of geographic atrophy (Sadda et al., 2018), chronic ocular hypertension (Bracha et al., 2018), as well as non-infectious intraocular inflammation and retinal vasculitis (Busch et al., 2022).

At present, medical treatment for DR is only applicable to patients with proliferative DR or diabetic maculopathy, but not to patients with less advanced DR (Mounirou et al., 2022). It is, however, important to note that diabetes-induced damage to the retinal neural cells and visual function, which may not be revealed by conventional fundus assessments, cannot be readily reversed. Given the immense associated socio-economical burdens, it is important to develop new treatment regimens to arrest the development of DR and prevent it from progressing to its proliferative stage in order to preserve vision.

Diabetes and its related complications are often associated with oxidative stress and a compromised antioxidant defense system (Jha et al., 2016). Furthermore, high metabolic activity and constant exposure to damaging light radiation may render the retina more vulnerable to oxidative stress under diabetic conditions. Therefore, there is growing interest in using antioxidants to treat DR. Our previous studies have demonstrated the involvement of mitochondrial dysfunction in DR using both *in vitro* and *in vivo* models (Lam et al., 2022, 2023). Similar findings have also been observed in other retinal cell types, such as retinal endothelial cells and Müller cells under simulated hyperglycemia (Trudeau et al., 2010; Tien et al., 2017). Thus, Coenzyme Q10 (CoQ10), a natural antioxidant with mitochondrial stabilizing properties, may be considered as a candidate for prophylaxis against DR.

CoQ10 is an essential endogenous enzyme cofactor of the electron transport chain, which exerts a protective effect on various cell types. It has been shown to reduce oxidative levels (Modi et al., 2006), improve insulin sensitivity (Kowluru and Mishra, 2015), and ameliorate diabetes-induced nephropathy (Ahmadvand et al., 2012), peripheral neuropathy (Zhang et al., 2013), and brain damage (Moreira et al., 2005) in rodent models of diabetes. It has also been found to protect neuronal cells in neurodegenerative diseases by stabilizing the mitochondrial membrane potential, supporting ATP synthesis, and inhibiting reactive oxygen species (ROS) generation (Shults et al., 2002). In addition, CoQ10 supplementation is associated with improved endothelial function in patients with atherosclerosis (Gao et al., 2012) and been demonstrated to protect the retina against oxidative stress and ischemic injury *in vitro* and *in vivo* (Nakajima et al., 2008; Lee et al., 2014).

CoQ10 is conventionally administrated as an oral supplement. However, the large molecular size and hydrophobic nature of CoQ10 constitute a major obstacle in achieving a reasonable bioavailability at the retina. This may explain the results obtained from two previous clinical trials, in which a significant improvement of best-corrected visual acuity was not observed, despite reduced serum ROS levels in non-proliferative DR patients taking oral CoQ10 supplement (Domanico et al., 2015; Rodríguez-Carrizalez et al., 2016).

This issue has been overcome by reformulation of CoQ10 into eyedrops with vitamin E d-α-tocopheryl poly(ethylene glycol) 1000 succinate (TPGS). The development of topical formulations of CoQ10 has been limited due to its low aqueous solubility and the presence of multi-drug efflux pump P-glycoprotein in corneal epithelial cells, which restricts drug absorption via corneal topical application (Vellonen et al., 2010). Vitamin E TPGS is a water-soluble derivative of vitamin E and a potent P-glycoprotein inhibitor (Yang et al., 2018). By conjugating CoQ10 with vitamin E TPGS, CoQ10 levels have been reported to increase significantly in the vitreous humor of patients following vitrectomy (Fato et al., 2010). Previous studies have demonstrated that topical application of this formulation allow penetration of CoQ10 to the retina (Fato et al., 2010; Lulli et al., 2012).

Previously, a study using short-chain quinones has shown improved visual acuity (measured with optokinetic response) and gliosis, as well as preserved ganglion cell count and total retinal thickness in a type 2 diabetic rat model (Daniel et al., 2021). However, the effect of such mitoprotective therapy on other retinal neural cells under diabetic condition is not well understood. The current study aimed to investigate the protective effect of CoQ10 against diabetic neuro-retinopathy in C57BLKsJ-db/db (db/db) mice, a type 2 diabetes model that possesses similar characteristics of retinal neurodegeneration to those of diabetic patients (Bogdanov et al., 2014). The study aimed to elucidate the effect of topical application of CoQ10 eyedrops (in conjunction with vitamin E TPGS) on different retinal neural cell types with a comprehensive electroretinography (ERG) protocol, which allowed a better evaluation of the electrophysiological changes of various retinal neural cells, together with immunohistochemistry for the structural assessment.

## 2 Materials and methods

### 2.1 Animal handling and CoQ10 treatment

Male C57BL/KsJ-db/db mice, obtained from The Chinese University of Hong Kong, were housed in the Central Animal Facility of The Hong Kong Polytechnic University. The mice were housed in groups of two in plastic cages with free access to standard rodent diet and water under a 12 h/12 h light-dark cycle. The experimental procedures were performed according to the ARVO Statement for the Use of Animals in Ophthalmic and Vision Research and were approved by the Animal Subjects Ethics Sub-committee (ASESC) of The Hong Kong Polytechnic University (approval no.: 19-20/85-SO-R-STUDENT).

Fasting blood glucose levels were measured monthly from baseline to 4-month after commencing the experiment with a digital blood glucometer (Accu-Chek^®^ Performa, Roche Diagnostics, Basel, Switzerland). The hemoglobin A1c (HbA1c) levels, which reflect the diabetic status over the prior three to four months, were measured at baseline and at 4-month after commencing CoQ10 treatment using DCA^®^ Vantage Systems (Siemens Medical Solutions Diagnostics, New York, NY, USA). Blood samples were collected from the tail veins after overnight fasting. All assessments were conducted between 9 am to 10 am. Bodyweight was monitored weekly. Food and water consumption were measured thrice weekly.

Mice with a fasting blood glucose level ≥ 13.9 mmol/L at 9-week of age were included in the study. The diabetic mice were divided into two groups, group 1 mice (CoQ10 treated) received one drop (approximately 30 μl) of CoQun^®^ (0.1% CoQ10 and 0.5% vitamin-E TPGS in buffered isotonic solution, VISUfarma, Roma, Italy) eyedrops topically twice a day for four months, while group 2 mice (db/db control) received the same amount of phosphate-buffered saline (PBS). Following the eyedrops application, the animals were kept restrained for about 20 s to allow absorption of the eyedrops. The remaining volume was gently swiped off at the canthus.

The fasting blood glucose levels, HbA1c levels, body weight (Supplementary Figure 1), and total consumption of food and water (Supplementary Table 1) of the db/db mice in control and treatment groups did not differ statistically throughout the experiment, implying CoQ10 eyedrop treatment had a minimal effect on the glycemic status of db/db mice.

### 2.2 Electroretinography (ERG)

The *in vivo* retinal functions were assessed with full field ERGs (ffERGs) at baseline, 1-, 2-, and 4-month after CoQ10 treatment as described previously (Lam et al., 2023). For scotopic ERG, the mice were dark-adapted overnight (at least 16 h) prior to testing in the experimental room. The animals were anesthetized with a weight-based intraperitoneal injection of Ketamine 100 mg/ml (Alfasan International BV, Woerden, Holland) and Xylazine 20 mg/ml (Alfasan International BV). The corneas of both eyes were anesthetized with Proxymetacaine 0.5% (Provain-POS^®^, Ursapharm, Saarbrücken, Germany) and maintained moist by a drop of 3% Carbomer 974P gel (Lacryvisc^®^, Alcon, France). The pupils were dilated using a drop of solution mixed with Tropicamide 0.5% and Phenylephrine 0.5% (Mydrin^®^-P, Santen, Osaka, Japan). The animals were then placed on a warming table maintained at 37°C. A gold ring electrode was placed in contact with the cornea as the active electrode. Two platinum needle electrodes were inserted subcutaneously at the base of the tail and the forehead as the ground and reference electrodes, respectively. The impedance of the active and reference electrodes was less than 10 kΩ.

Visual stimuli with white light-emitting diodes were delivered by a Ganzfeld bowl (Q450, Roland Consult, Brandenburg, Germany). Stimulation and data recording were performed using the RETI-Port system^®^ (Roland Consult) according to a customized protocol, with stimulus intensities varying from −4.32 log cd s/ m^2^ to +1.3 log cd s/ m^2^. The signals were amplified and band-pass filtered from 1 to 30 Hz and 1 to 1,000 Hz for scotopic threshold response (STR) and scotopic a- and b-wave, respectively. At the lowest intensity, 40 sweeps of response were averaged with a stimulus frequency of 0.5 Hz. The number of sweeps and stimulus frequency was reduced at higher flash intensity levels. The stimulus intensities were calibrated by a photometer (ILT1700, International Light, MA) and converted to unit photoisomerizations/rod (R*/rod), where 1 scot cd/ m^2^ = 516 R*/rod/s. The stimulus responses within operational range of the rod cells (< −0.3 log cd s / m^2^) were fitted with a sigmoidal curve using the Naka-Ruston equation, a saturating hyperbolic function that describes the relationship between b-wave amplitude and flash intensity, to determine the maximum b-wave response (Bmax).

Oscillatory potentials (OPs) were isolated by digital filtering of the raw signal recorded at +1.3 log cd s/m^2^ using a fast Fourier transform (FFT) and subsequent inverse FFTs with an algorithm computed in the free software environment R (v4.0.4). The raw data was first converted from the time domain to the frequency domain. Spectral components beyond the cut-off frequency (65 Hz to 300 Hz) were then eliminated. The inverse FFT was performed to reconstruct the OP waveform in the time domain. Implicit times of OP were measured from the stimulus onset to the peak of the OP. Individual OP amplitudes were measured from the peak to the adjacent trough. The first four major OP wavelets were included for analysis.

Photopic ERG was conducted in a subset of animals on a separate day (without dark adaptation before the test) using the same system. The animals were presented to a uniform white background of 30 cd/ m^2^, and white light-emitting diodes flash (+0.47 log cd s/m^2^) was used as the stimulus. The signals were amplified and band-pass filtered from 1 to 1,000 Hz, and 40 sweeps of response were averaged with a stimulus frequency of 0.5 Hz. The amplitude and implicit time of b-wave were used for analysis.

### 2.3 Retinal immunohistochemistry (IHC)

At the end of the study period, one eye from each animal (randomly chosen) was enucleated and dissected to isolate the whole retina, which was then fixed in 4% paraformaldehyde in PBS at room temperature for one hour. Part of the harvested retinas (whole-mount) were used to quantify microglial density, while the remainder were processed into vertical retinal sections (35 μm) using a microtome (Vibratome VT1200S, Leica Microsystems, Wetzlar, Germany) as previously described (Tse et al., 2015). These samples were then blocked with 10% donkey serum in TBS (0.5% Triton X-100 and 0.1% Sodium Azide in Dulbecco’s phosphate-buffered saline, pH 7.2) at 4°C overnight to reduce nonspecific labeling.

The samples were then incubated with primary antibodies at 4°C in TBS with 3% donkey serum for four days. Following incubation, the samples were washed several times and transferred to a 3% normal donkey serum-TBS solution containing donkey-host secondary antibodies at 4°C overnight. DAPI (Invitrogen, D1306) was used to stain the nuclei in the retina. The details of antibodies used are listed in Supplementary Table 2.

A confocal laser scanning microscope (LSM800, Zeiss, Oberkochen, Germany) was used to capture confocal micrographs of the specimens using a 20×, 40×, or 63× objective. To determine the number of photoreceptor cells (including both rods and cones), the DAPI stained nuclei in the outer nuclear layer were counted over an 80 μm-segment with a cell counter in ImageJ (v1.53k, National Institutes of Health, Bethesda, MD, USA). To measure the number of cone photoreceptors and rod bipolar cells (and their synaptic terminals), the G Protein Subunit Alpha Transducin 2 (GNAT2) and Protein kinase C-α (PKCα) positive cells were counted over a 160 μm-segment. The number of rod-bipolar dendritic boutons was determined by counting the number of PKCα positive puncta at the outer plexiform layer over a 20 μm-segment. The dimensions of the outer nuclear layer and the inner nuclear layer were analyzed by ImageJ. The immunoreactivity of the vertical retinal sections against glial fibrillary acidic protein (GFAP) antibody was used to reflect the levels of glial reactivity in the retina, which was quantified based on the extent of GFAP staining using a scoring system described previously (Anderson et al., 2008; Bogdanov et al., 2014). The data of each individual animal was averaged from three retinal sections, with each group consisting of at least five animals. For quantification of microglia, the flat retinas were processed with ionized calcium binding adaptor molecule-1 (iba-1) antibody. Four regions of each flat retina around one disk diameter from the optic nerve head and two retinal regions (i.e., ganglion cell layer to inner plexiform layer and outer plexiform layer) were imaged with a 20× objective. The number of microglia (iba-1 positive cells) was counted with the cell counter in ImageJ. The data of each individual animal was averaged from the four regions per retina, each group consisting of at least four animals.

### 2.4 Evaluation of matrix metallopeptidase-9 (MMP-9) levels

A semi-quantitative analysis approach was used to assess the MMP-9 levels in the retina of the mice in the two groups due to the limited number of samples from this longitudinal study. The MMP-9 levels were evaluated by quantifying the fluorescence intensity levels in the retinal sections after immunostaining with ImageJ. The images were first converted to gray scale. The mean fluorescence was measured across the whole retinal section imaged, and the value subtracted from that of the background. The data of each individual animal was averaged from three retinal sections, each group consisting of at least five animals. To ensure the specificity of the antibody, retinal sections of a wildtype mouse were processed using the same procedure, but omitting the primary antibody. Only trace fluorescence signals were detected in the negative control confocal images.

### 2.5 Measurement of retinal mitochondrial bioenergetics

The mitochondrial bioenergetics of the retinas of the db/db mice in both groups were assessed by measuring the oxygen consumption rate (OCR) with a Seahorse XFe24 Extracellular Flux Analyzer (Agilent Technologies, Santa Clara, CA, USA). Four mice were chosen randomly from each of the db/db treated and db/db control groups. One eye from each animal was enucleated and dissected to harvest the whole-mount retina in ice-cold PBS. Three 1.5 mm diameter punches were obtained from the neural retina using a biopsy puncher (Miltes Instrument, Integra LifeSciences, Mansfield, MA, USA). The retinal punches were obtained adjacent to the optic nerve head to minimize any discrepancy in cell density. Each retinal punch was then carefully placed in the center of the well of an XF24 Islet capture microplate (Agilent Technologies), with the ganglion cell layer facing up and then covered with Islet Fluxpak mesh inserts. Prior to the measurement of OCR, Seahorse XF DMEM medium (103335-100, Agilent Technologies) containing 5.5 mM glucose (G6152, Sigma-Aldrich) and 1 mM sodium pyruvate (11360070, Gibco) was added to each well and the retinal punches incubated at 37°C in a non-CO^2^ incubator for one hour to allow the temperature and pH to reach equilibrium. The OCR was then measured under basal conditions and after serial injection of 1 μM carbonyl cyanide-4-(trifluoro-methoxy) phenylhydrazone (FCCP; 15218, Cayman), and a mixture of 10 μM rotenone (13995, Cayman) and 20 μM antimycin A (A8674, Sigma-Aldrich, St. Louis, MO, USA) to determine values for basal respiration, maximal respiration, spare respiratory capacity, and non-mitochondrial oxygen consumption. Following the assays, the retinal punches in each well were lysed with EB2 lysis buffer, containing 7 M urea, 2 M thiourea, 30 mM Tris, 2% (w/v) CHAPS, and 1% (w/v) ASB14 with protease inhibitor cocktail (Roche Applied Science, Basel, Switzerland), and placed on ice. The protein concentrations were determined using the Bradford Protein Assay (Bio-Rad Laboratories Inc., St. Louis, MO, USA) according to the manufacturer’s guidelines for normalization of the OCR values. The values were then normalized to those of the control mice.

### 2.6 Statistical analysis

Data are presented as mean ± SEM. Shapiro–Wilk test was used to ensure the data was normally distributed prior to the use of a parametric test. For longitudinal comparison, mixed-model ANOVA was used to analyze the within-group, between-group, and interactive effects. Independent sample *t*-test (with Welch correction when appropriate) was used for comparison between the two groups. JASP (v0.13, Amsterdam, Netherlands) was used for the statistical analysis. Differences with a *p*-value < 0.05 were considered significant.

## 3 Results

### 3.1 CoQ10 eyedrops conjugated with vitamin E TPGS preserved retinal functions in db/db mice

**FIGURE 1:**
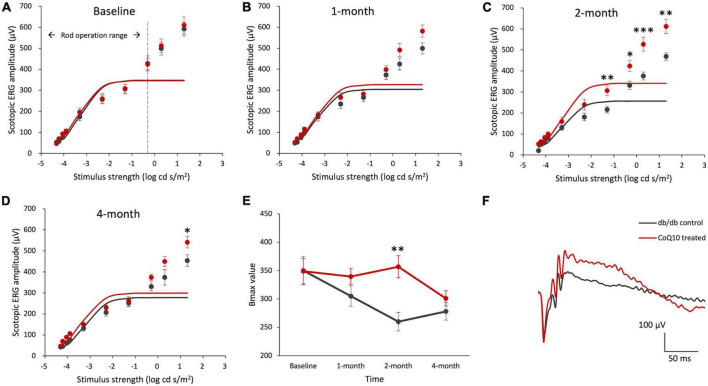
Effect of CoQ10 eyedrops conjugated with vitamin E TPGS on scotopic ERG b-wave at different experimental timepoints. **(A–D)** Stimulus-response plots showing the amplitude of scotopic ERG pSTR (–4.32 to –3.9 log cd s /m^2^) and b-wave response (–3.3 to +1.3 log cd s /m^2^) recorded from control db/db mice (*n* = 11) and CoQ10 treated mice (*n* = 12) at different experimental timepoints. The responses within the rod operative range (i.e., ≤ –0.3 log cd s /m^2^) were fitted with sigmoidal curves using the Naka-Ruston function to deduce the maximum b-wave response (Bmax) value. **(E)** The Bmax value of the two groups of mice over the experimental period (Data presented as mean ± SEM. Simple main effect analysis: *p < 0.05, **p < 0.01, ***p < 0.001). **(F)** Representative scotopic ERG waveform at +1.3 log cd s/m^2^ of the db/db control mice (gray) and CoQ10 treated mice (red) at 4-month.

The effect of CoQ10 treatment on preserving the function of different retinal types against diabetes was assessed by ffERGs. The scotopic a-wave reflects the photoreceptor cell function, particularly that of rod cells. The amplitude and implicit time of scotopic a-wave were not statistically different between the control and CoQ10 treated groups at any of the experimental timepoints (Supplementary Figure 2). In contrast, the scotopic b-wave, an indicator of the functions of the rod cell and its post-receptoral pathway, was significantly stronger in the CoQ10 treated group than the control group at some of the tested stimulus intensities (Figure 1; see Supplementary Figure 3 for the scotopic b-wave implicit time).

The maximum rod-driven b-wave response amplitude (Bmax) was deduced by fitting the ERG responses within the rod operation range (i.e., < −0.3 log cd s / m^2^) with sigmoidal curves using the Naka-Ruston function (Figures 1A–D). Repeated measures ANOVA revealed a significant main effect of time and groups, as well as a significant interactive effect between time and groups (Repeated measures ANOVA, time: *F* = 5.304, df = 3, *p* = 0.003; group: *F* = 4.559, df = 1, *p* = 0.045; time × group: *F* = 3.630, df = 3, *p* = 0.018). At baseline, the difference in the Bmax value was not statistically significant between the two groups of mice (Figure 1E). After two months of CoQ10 treatment, the Bmax value of the CoQ10 treated db/db mice became significantly stronger than that of the control mice. This effect, however, was not sustained at 4-month.

**FIGURE 2:**
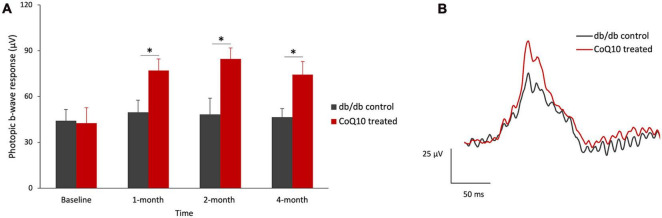
Effect of CoQ10 eyedrops conjugated vitamin E TPGS on photopic ERG b-wave at different experimental timepoints. **(A)** The photopic ERG responses of the control (*n* = 5) and CoQ10 treated db/db mice (*n* = 5) were measured at +0.47 log cd s/m^2^ (Data presented as means ± SEM. Simple main effect analysis: *p < 0.05). **(B)** Representative photopic ERG waveform at +0.47 log cd s/m^2^ of control mice (gray) and CoQ10 treated mice (red) at 4-month.

Intriguingly, a significantly stronger b-wave response was observed in the CoQ10 treated group at +1.3 log cd s/m^2^, in which the response was driven by both rod and cone cells, after two months of CoQ10 treatment compared to the control groups (2-month: db/db control = 468.75 ± 17.51 μV vs. CoQ10 treated = 611.79 ± 33.71 μV; Simple main effect analysis, *p* = 0.001; 4-month: db/db control = 454.02 ± 27.06 μV vs. CoQ10 treated = 541.11 ± 27.12 μV; Simple main effect analysis, *p* = 0.034). The representative ERG waveforms of the two groups of mice recorded at this intensity at 4-month are shown in Figure 1F. This suggests that the CoQ10 treatment may have preserved the functions of the cone photoreceptors and/ or their related postsynaptic pathway. In agreement, it was observed that CoQ10 treated db/db mice exhibited a significantly stronger photopic b-wave amplitude after receiving CoQ10 eyedrops for one month (Figure 2, Mixed model ANOVA, time: *F* = 3.093, df = 3, *p* = 0.046; group: *F* = 16.809, df = 1, *p* = 0.003; time × group: *F* = 1.986, df = 3, *p* = 0.143), though the implicit time was not significantly different (Supplementary Figure 4). The results further supported the protective effect of CoQ10 eyedrops on the cone-related pathway.

**FIGURE 3:**
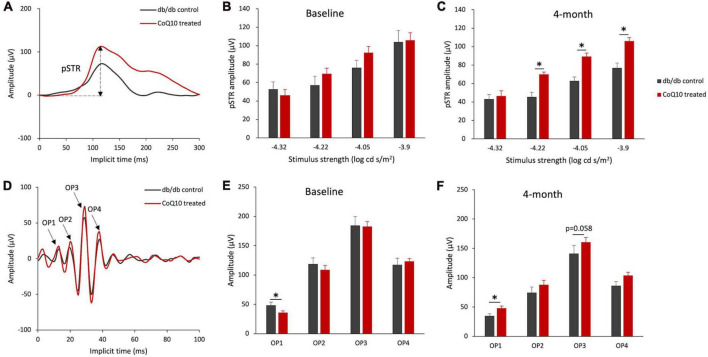
Effect of CoQ10 eyedrops conjugated vitamin E TPGS on inner retinal function at different experimental timepoints. **(A)** Representative waveform showing scotopic threshold response (STR) at –3.9 log cd s/m^2^ of the db/db control mice (gray) and CoQ10 treated mice (red) at 4-month. Arrow shows the positive STR (pSTR) of the CoQ10 treated db/db mice. **(B,C)** Bar charts showing the mean amplitude of pSTR measured at different stimulus intensities of the control db/db mice (*n* = 11) and CoQ10 treated mice (*n* = 12) at baseline and 4-month. The oscillatory potentials (OPs) were isolated by digitally filtering the raw ERG signals recorded at +1.3 log cd s/m^2^. **(D)** The representative OPs waveforms of the db/db mice in control (gray) and CoQ10 treated (red) group at 4-month. Arrows indicating the peaks of OP1 to OP4 of the CoQ10 treated db/db mice. **(E,F)** Bar charts showing the mean amplitudes of OP1 to OP4 and of the control mice (*n* = 11) and CoQ10 treated mice (*n* = 12) at baseline and 4-month (Data presented as mean ± SEM. Simple main effect analysis: *p < 0.05).

For the inner retinal function, the positive scotopic threshold response (pSTR) and oscillatory potentials (OPs), which are presumed to originate from the ganglion cells (Saszik et al., 2002) and amacrine cells (Wachtmeister, 1998), respectively, were analyzed. The representative pSTR waveforms of the two groups of mice at 4-month are shown in Figure 3A. The results revealed that the amplitude of pSTR was not statistically different between the two groups of mice (Figure 3B) but became significantly stronger in the CoQ10-treated group than the control group after 4 months of CoQ10 treatment, indicating preserved ganglion cell function (Figure 3C, please refer to Supplementary Figure 5 for the implicit time of pSTR). The OPs were obtained by digitally filtering the raw ERG signals recorded at +1.3 log cd s/m^2^ (see Figure 3D for the representative OPs waveforms). At baseline, the control db/db mice were found to have a significantly stronger OP1 response amplitude (Figure 3E, db/db control = 48.72 ± 5.16 μV vs. CoQ10 treated = 36.17 ± 2.63 μV; Simple main effect analysis, *p* = 0.037). In contrast, the db/db mice in the treatment group exhibited stronger OP1 amplitude compared to their diabetic control littermates after 4 months of CoQ10 treatment (Figure 3F, db/db control = 35.13 ± 3.32 μV vs. CoQ10 treated = 48.14 ± 3.66 μV; Simple main effect analysis, *p* = 0.016). Implicit times of OP1-OP4 were not significantly different between the two groups of mice at baseline and 4-month (Supplementary Figure 6).

### 3.2 CoQ10 eyedrops conjugated with vitamin E TPGS preserved the retinal structure of the db/db mice

**FIGURE 4:**
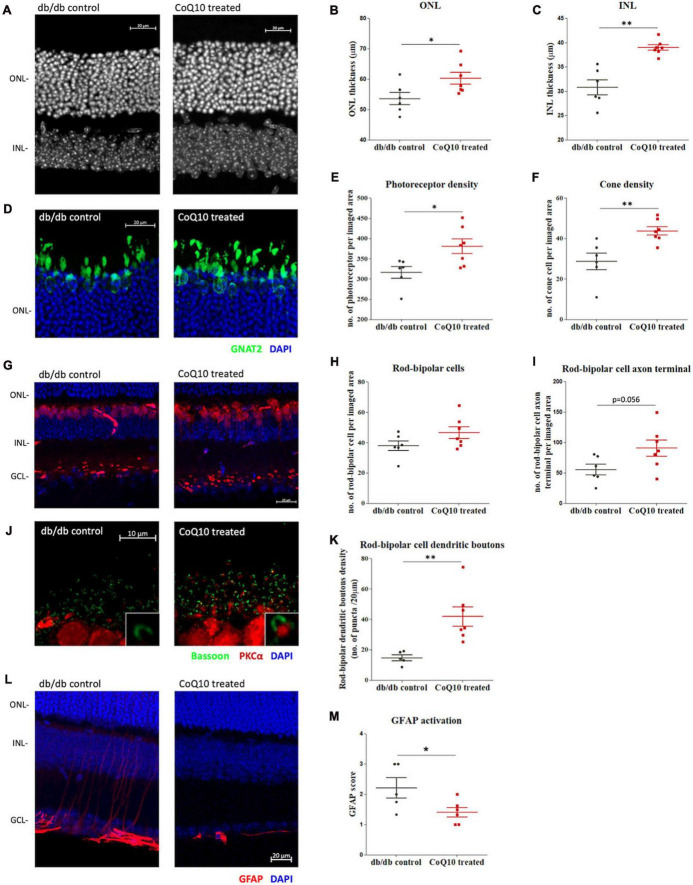
The effect of CoQ10 eyedrops conjugated vitamin E TPGS on preserving the retinal structure of db/db mice. **(A)** Representative confocal images of the retinal sections of the two groups of mice processed for DAPI (white). White scale bar: 20 μm. Dot plots comparing the thickness of the **(B)** outer nuclear layer (ONL) and **(C)** inner nuclear layer (INL) of the control db/db mice (*n* = 6) and CoQ10 treated mice (*n* = 7). **(D)** Representative confocal images of the retinal sections focusing on the ONL of the two groups of mice. Bar charts comparing the number of **(E)** photoreceptors and **(F)** cone cells of the control db/db mice (*n* = 6) and CoQ10 treated mice (*n* = 7). The number of photoreceptors was assessed by counting the DAPI stained nuclei (blue), and the number of cones by counting the soma co-labeled by GNAT2 (green) over an 80 μm- and a 160 μm-segment, respectively. **(G)** Representative confocal images of the retinal sections of the control db/db mice and CoQ10 treated db/db mice processed for PKCα (red) and DAPI (blue). White scale bar: 20 μm. Dot plots comparing the **(H)** rod bipolar cell density and **(I)** number of rod bipolar cell axon terminals of control (*n* = 6) and CoQ10 treated db/db mice (*n* = 7). **(J)** Representative confocal images of the retina sections of the control and CoQ10 treated mice processed for PKCα (red) and bassoon (green). White scale bar: 10 μm. **(K)** Dot plots comparing the rod bipolar cell dendritic boutons of control (*n* = 5) and CoQ10 treated db/db mice (*n* = 7). **(L)** The representative confocal images of the retinal sections processed with GFAP (red), which reflects the extend of glial reactivity from the two groups of mice. White scale bar: 20 μm. **(M)** Dot plots showing the GFAP score of the control (*n* = 5) and CoQ10 treated mice (*n* = 6) (Data presented as mean ± SEM. Independent sample *t*-test: **p < 0.05). ONL, outer nuclear layer; INL, inner nuclear layer; GCL, ganglion cell layer.

At the experimental endpoint, one eye from the animal was randomly chosen and processed for immunohistochemistry analysis as whole mount or microtome-sectioned retina. Results showed that CoQ10 treated db/db mice had a significantly thicker ONL (db/db control = 53.60 ± 2.03 μm vs. CoQ10 treated = 60.26 ± 1.96 μm; Independent sample *t*-test: *t* = −2.357, df = 11, *p* = 0.038) and INL (db/db control = 30.85 ± 1.55 μm vs. CoQ10 treated = 39.03 ± 0.58 μm; Independent sample *t*-test (Welch): *t* = −4.9553, df = 6.379, *p* = 0.002) compared to the db/db control mice (Figures 4A–C).

The photoreceptor cell density (DAPI-stained cells in theONL) of CoQ10 treated db/db mice was found to be significantlyhigher than that of the control db/db mice (db/db control = 316.42 ± 14.35 vs. CoQ10 treated = 380.81 ± 17.97; Independent sample *t*-test: *t* = −2.733, df = 11, *p* = 0.019). In addition, CoQ10 treated db/db mice had more cone soma (nucleus co-stained with GNAT2) then the control db/db mice (db/db control = 28.76 ± 4.10 vs. CoQ10 treated = 43.81 ± 2.09; Independent sample *t*-test: *t* = −3.422, df = 11, *p* = 0.006) (Figures 4D-F).

The rod-bipolar cells were examined by immunostaining with antibodies against PKCα (Figure 4G). Although the rod bipolar cell density of the CoQ10 treated db/db mice was higher than that of the control mice, the difference did not reach statistical significance (Figure 4H, db/db control = 38.01 ± 3.21 vs. CoQ10 treated = 46.60 ± 3.83; Independent sample *t*-test: *t* = −1.682, df = 11, *p* = 0.121). Previously, we reported a reduced number of rod-bipolar cell axon terminals and dendritic boutons in db/db mice, indicating that diabetes leads to a compromised synaptic connectivity of the second-order neurons with other retinal neurons (Lam et al., 2023). Based on these earlier findings, the number of rod-bipolar cell axon terminals and dendritic boutons in the db/db mice after CoQ10 treatment were examined. The number of rod-bipolar cell axon terminals tended to be higher in CoQ10 treated db/db mice than the control mice. However, the difference did not reach statistical significance (Figure 4I, db/db control = 55.75 ± 8.75 vs. CoQ10 treated = 90.93 ± 13.19; Independent sample *t*-test: *t* = −2.14, df = 11, *p* = 0.056). In contrast, the density of rod-bipolar cell dendritic boutons (Figure 4J) in the CoQ10 treated db/db mice was significantly higher than the db/db control mice (Figure 4K, db/db control = 14.69 ± 1.91 vs. CoQ10 treated = 41.85 ± 6.35; Independent sample *t*-test: *t* = −3.488, df = 10, *p* = 0.006), suggesting an ameliorated loss of synaptic connectivity, particularly, between photoreceptor and the second-order neurons.

Glial reactivity (gliosis) in the retina is associated with early neuronal impairment of diabetic retinopathy (Sorrentino et al., 2016). The effect of CoQ10 eyedrops conjugated with vitamin E TPGS on gliosis was investigated by processing the vertical retinal sections with GFAP antibody (Figures 4L, M), which is known to be expressed in astrocytes and reactive Müller cells (Hol and Pekny, 2015; Lee et al., 2021). The extent of gliosis was determined by a scoring system described previously (Anderson et al., 2008; Bogdanov et al., 2014). The results showed that CoQ10 eyedrops conjugated with vitamin E TPGS could significantly reduce gliosis in the retina of db/db mice (db/db control = 2.22 ± 0.34 vs. CoQ10 treated = 1.41 ± 0.16; Independent sample *t*-test: *t* = 2.302, df = 9, *p* = 0.047).

**FIGURE 5:**
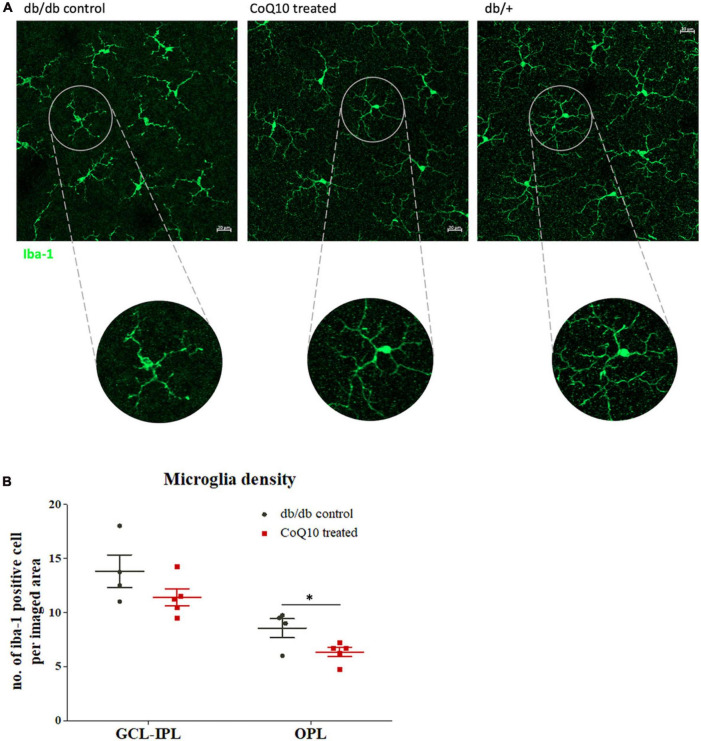
The effect of CoQ10 eyedrops conjugated vitamin E TPGS on retinal microglial cells in db/db mice. **(A)** Representative confocal images of the flat mount retina of the db/db mice in the treatment and control group processed for iba-1 (green) with that of heterozygous non-diabetic control (db/+ mice) included as reference. The morphology of the iba-1 positive cells in the CoQ10 treated group appeared to be more ramified than that of the db/db control. White scale bar: 20 μm. **(B)** Dot plots comparing the number of iba-1 positive cell per imaged area of the control db/db mice (*n* = 4) and CoQ10 treated mice (*n* = 5) at GCL-IPL and OPL (Data presented as mean ± SEM. Independent sample *t*-test: *p < 0.05). OPL, outer plexiform layer; IPL, inner plexiform layer; GCL, ganglion cell layer.

Microglia are the principal resident immune cell in the retina, playing an important role in immune surveillance and synapse maintenance (Wang et al., 2016). Increase in microglial density and changes in morphology have been implicated in a range of degenerative eye diseases (Fan et al., 2022). The distribution of the microglia at two retinal regions [i.e., ganglion cell layer to inner plexiform layer (GCL-IPL) and outter plexiform layer (OPL)] was studied. Results showed that retinas of db/db mice receiving CoQ10 eyedrops conjugated with vitamin E TPGS had reduced microglial density in the OPL (db/db control = 8.56 ± 0.87 vs. CoQ10 treated = 6.35 ± 0.43; Independent sample *t*-test: *t* = 2.444, df = 7, *p* = 0.044) but not in the GCL-IPL when compared to the db/db control mice (db/db control = 13.81 ± 1.51 vs. CoQ10 treated = 11.40 ± 0.79; Independent sample *t*-test: *t* = 1.509, df = 7, *p* = 0.175). In addition, the microglial cell in the retinas of the control group appeared to be less ramified than those in the treatment group (Figure 5).

### 3.3 CoQ10 eyedrops conjugated with vitamin E TPGS improves retinal mitochondrial bioenergetics of db/db mice while alleviating retinal levels of MMP-9

**FIGURE 6:**
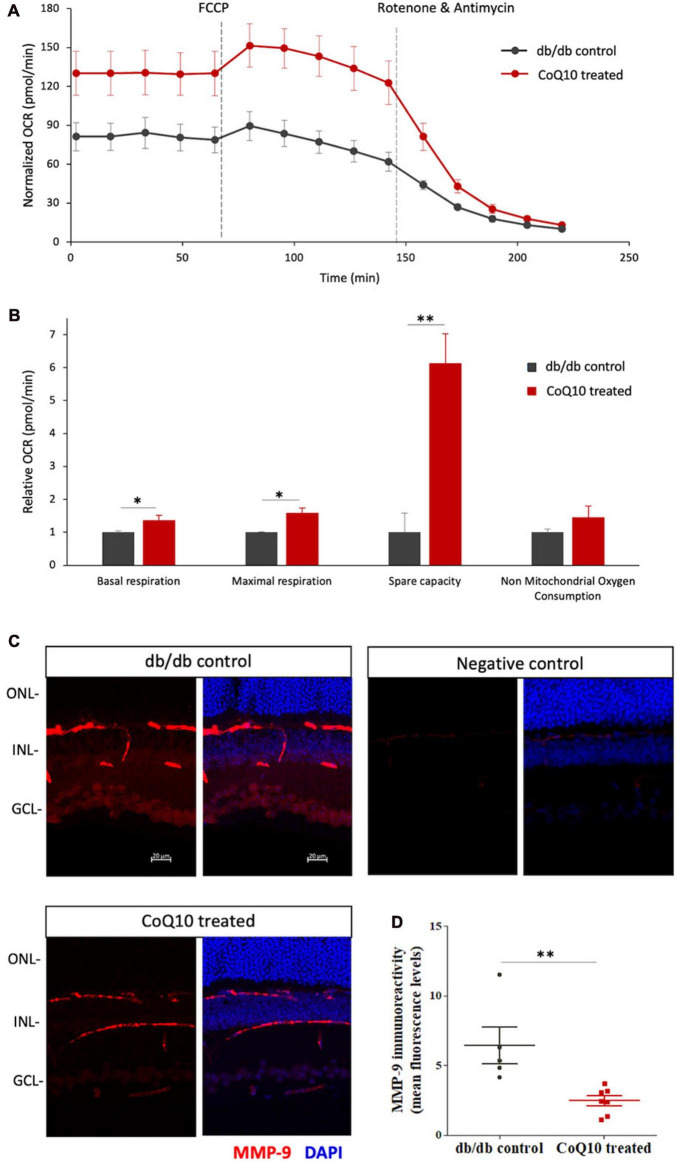
The effect of CoQ10 eyedrops conjugated vitamin E TPGS on mitochondrial bioenergetics and MMP-9 levels of the retinas of db/db mice. **(A)** The oxygen consumption rate (OCR) curve measured from the retina of the two groups of mice with the Seahorse Mito Stress assay. The value was normalized to the protein concentration. **(B)** Bar charts comparing the key mitochondrial function parameters of the control (*n* = 4) and CoQ10 treated mice (*n* = 4) (Data presented as means ± SEM. Independent sample *t*-test, *p < 0.05, **p < 0.01). **(C)** Representative confocal images of the retinal sections processed with MMP-9 (red). DAPI (blue) was used to counterstain the cell nucleus to indicate different retinal layers of the two groups of mice. Negative control retinal sections (primary antibody omitted) from wild type mice show only trace fluorescence. White scale bar: 20 μm. **(D)** Dot plots showing the mean fluorescence levels of the images, which reflect the levels of MMP-9 in the retina of the control (*n* = 5) and CoQ10 treated mice (*n* = 7). MMP-9 has been detected in various retinal cell types, including vascular cells, glial cells, and neural cells, in different disease models. The immunoreactivity of MMP-9 was found more prominent in vessel, inner nuclear layer, inner plexiform layer, and ganglion cell layer in db/db mice retina, while CoQ10 eyedrops conjugated with vitamin E TPGS attenuated the increase in fluorescence intensity (Data presented as means ± SEM. Independent sample *t*-test, *p < 0.05). ONL, outer nuclear layer; INL, inner nuclear layer; GCL, ganglion cell layer.

The effect of CoQ10 on the mitochondrial bioenergetics of the *ex-vivo* retina of the db/db mice in the treatment and control group was tested after four months of CoQ10 treatment using a Seahorse XF Analyzer. Compared to the control mice, CoQ10 treated db/db mice exhibited a significantly higher basal respiration (1.373 ± 0.142-fold, *p* = 0.044), maximal respiration (1.589 ± 0.154-fold, *p* = 0.031), and spare capacity (6.133 ± 0.895-fold, *p* = 0.003). These results indicate a stronger mitochondrial function in the retina of db/db mice treated with CoQ10 compared to db/db control mice (Figures 6A, B).

The ameliorated retinal mitochondrial bioenergetics of the CoQ10 treated db/db mice were accompanied by an alleviated immunoreactivity of MMP-9 (Figures 6C, D). Results from immunohistochemistry showed that the retinal sections of CoQ10 treated db/db mice had a significantly lower immunoreactivity toward MMP-9 compared to the control db/db mice (db/db control = 6.44 ± 1.32 vs. CoQ10 treated = 2.49 ± 0.36; Independent sample *t*-test: *t* = 3.364, df = 10, *p* = 0.007).

## 4 Discussion

Mitochondrial dysfunction has emerged as a critical pathway underlying the development of diabetes and associated complications. Mitochondrial dynamics and function may affect the survival of retinal neural and glial cells through the modulation of neurosteroids and sigma receptor 1 signaling in disease conditions (Bucolo and Drago, 2004; Bucolo et al., 2006; Zhou et al., 2020). Defective mitochondrial biogenesis and changes in energy metabolic profile have been implicated in various cell types and tissues affected by type 2 diabetes (Jheng et al., 2012; Forbes and Thorburn, 2018; Pinti et al., 2019). In addition, mitochondrial dysfunction has been observed in various retinal cells, including retinal endothelial cells, Müller cells, and photoreceptor cells under simulated hyperglycemia (Trudeau et al., 2010; Tien et al., 2017; Lam et al., 2022). Recently, our lab has demonstrated a compromised mitochondrial biogenesis in the retina of db/db mice, in which functional and structural changes in the neuroretina were observed, compared to their non-diabetic heterozygous littermate (Lam et al., 2023). These findings suggested that mitoprotection may be beneficial in ameliorating DR.

Mitoprotective agents, elamipretide and short-chain quinones, have been shown to improve visual acuity (measured with optokinetic response) and gliosis, and preserve ganglion cell count and total retinal thickness in a previous study using a type 2 diabetic rat model (Daniel et al., 2021). Indeed, diabetes affect the entire neovascular unit of the retina (Sinclair and Schwartz, 2019). However, the effect of mitoprotective therapy on retinal neural cells, other than retinal ganglion cells, under diabetic condition remains elusive. The current study sought to investigate the protective effect of a topical form of CoQ10 (in conjunction with vitamin E TPGS) on diabetes-induced retinal neurodegeneration, in particular, the electrophysiological changes, with a comprehensive ERG protocol and immunohistochemistry, to elucidate its effect on different retinal neural cells. The effect of this topical formulation on mitochondrial biogenesis in the diabetic retina was also assessed *ex vivo*. The findings suggested that CoQ10 (in conjunction with vitamin E TPGS) could ameliorate the functional and structural changes of a range of retinal neural cells under diabetes and could be considered as a complementary treatment against DR.

The current study showed that db/db mice receiving CoQ10 eyedrops conjugated with vitamin E TPGS exhibited a stronger pSTR and OP1 amplitude compared to the control db/db mice, indicating a strong inner retinal function. An initial improvement in the outer retinal function was also observed in the CoQ10 treated db/db mice, in which a significantly stronger rod-driven b-wave maximum amplitude response was found compared to their age-matched diabetic controls after two months of treatment, although the effect was not sustained at 4-month. Interestingly, the ERG result revealed a preserved scotopic ERG b-wave response at +1.3 log cd s/m^2^ in CoQ10 treated db/db mice, in which the response was driven by both rod and cone cells. Thus, photopic ERG response, which directly reflects the function of the cone-related pathway, was measured. It was found that CoQ10 treated db/db mice has a significantly stronger photopic ERG response compared to the control db/db mice after receiving the CoQ10 eyedrops for one month. This suggested a protective effect of CoQ10 eyedrops on photoreceptor cone cells and their related postsynaptic pathway.

It has been suggested that elevated glucose levels could exacerbate cone metabolism and specifically render the cone photoreceptor cells more susceptible to oxidative stress in diabetes (Kurtenbach et al., 2006). Owing to the difference in metabolic demand and ability to maintain cellular homeostasis from rod photoreceptors (Okawa et al., 2008; Hutto et al., 2020), the protective effect of CoQ10 may be more pronounced in cone photoreceptors. This may render a high therapeutic value when applied to humans since the human retina has a higher cone to rod ratio (1:20) than the murine retina (1:35) (Jeon et al., 1998; Hussey et al., 2022). More importantly, the foveal region, which is responsible for the central vision of humans, consists only of cone cells.

The protective effect of CoQ10 against diabetic retinal neuropathy is further supported by the histological data, in which CoQ10 treated db/db mice were found to have thicker ONL and INL and higher photoreceptor and cone soma densities compared with their age-matched controls. Our lab and others have previously demonstrated disruption of synaptic structures in second-order neurons at the outer plexiform layer without apparent decreased bipolar cell viability in diabetic mice (Hombrebueno et al., 2014; Lam et al., 2023). In this study, the number of rod-bipolar cell dendritic boutons was found to be significantly preserved in CoQ10 treated db/db mice compared to the controls. This effect may be attributed to the enhanced retinal mitochondrial bioenergetics with CoQ10 treatment observed in this study. Additionally, retinas of db/db mice in the treatment group appeared to have less gliosis, a lower microglial density, and preserved microglial morphology.

CoQ10 is a well-known antioxidant and mitochondrial stabilizer. It has been found to improve bioenergetic function in isolated astrocytes from the optic nerve head, resulting in increased mitochondrial mass and attenuated oxidative stress and cell death (Noh et al., 2013). Apart from conserving the mitochondrial bioenergetic, the results also indicated that CoQ10 may protect the mitochondria via regulating the retinal levels of MMP-9 in diabetic mice. Activation of MMP-9 in the retina is one of the early event of DR, leading to mitochondrial damage and apoptosis (Kowluru et al., 2011; Duraisamy et al., 2017). Elevated expressions of MMPs have been suggested to increase vascular permeability via a mechanism involving proteolytic degradation of tight junction proteins (e.g., occludin) (Kowluru and Shan, 2017). In addition, enhanced MMP-9 expression observed in cerebral ischemia has been associated with excitotoxicity, neuronal damage, apoptosis, and blood-brain barrier breakdown, resulting in cerebral edema and haemorrhagic transformation (Chaturvedi and Kaczmarek, 2014). The reduced MMP-9 levels after topical application of CoQ10 with vitamin E TPGS suggest a beneficial effect to both neural and vascular components of the diabetic retina. MMP-9 has been shown to mediate hyperglycemia-induced ROS production in human cardiac stem cells (Yadav et al., 2020), thus a reduced level of MMP-9 may also imply a lower ROS level in the retina of the CoQ10 treated db/db mice in the current study.

The relationship between CoQ10 and the expression of MMP-9 in the diabetic retina is not explicit, but a previous study on radiation enteropathy demonstrated that CoQ10 could alleviate MMP-9 levels via transforming growth factor-beta (TGF-β) signaling (Mohamed and Said, 2021). TGF-β singaling has been implicated in the pathogenesis of diabetes and related complications, including DR (Betts-Obregon et al., 2016; Heydarpour et al., 2020). Diabetic patients have been found to have higher TGF-β1 serum levels than healthy controls, and their TGF-β1 serum levels have also been correlated with the severity of DR (Bonfiglio et al., 2020). In-depth exploration of how CoQ10 can moderate MMP-9/TGF-β singaling may provide further therapeutic insight for DR.

Nevertheless, the findings of the current study provide further insight into the role of mitochondrial dysfunction in the development of DR-related neurodegeneration and suggest that CoQ10 eyedrops conjugated with vitamin E TPGS may serve as a complementary therapy against DR-related neuropathy. Corneal topical application offers a simple and risk-free treatment method compared to other routes of administration, such as intravitreal injection (risk of developing endophthalmitis after intravitreal injection is about 0.056%) (Fileta et al., 2014). One limitation of this study is that we cannot conclude if the observed effect could be attributed to CoQ10 alone or if there is any synergic effect when co-administrated with vitamin E TPGS. Despite the wide application in enhancing drug delivery (Luiz et al., 2021), vitamin E TPGS has been shown to induce mitochondrial destabilization and successive activation of mitochondrial apoptotic mediators, specifically in many cancer types but not in normal cells and tissues (Neuzil et al., 2004, 2007). Although vitamin E TPGS does not exert antioxidative properties unless being decomposed by cellular esterases to liberate α-tocopherol (Yan et al., 2007), its effect on neural cells under diabetic stress remains elusive. In our study, only male mice were used since they display a more severe and uniform diabetes phenotype and are less affected by estrogen cycle (Daniels Gatward et al., 2021). However, incorporating female animals may enhance the translatability of the research outcome. It should also be noted that no apparent vascular change, a critical clinical hallmark of DR, was observed in the animal model within the relatively short experimental period. Further study is warranted to determine whether topical treatment of CoQ10 with vitamin E TPGS would have any beneficial effect on diabetic retinal vasculopathy.

## 5 Conclusion

Our findings suggest that topical application of CoQ10 eyedrops (conjugated with Vitamin E TPGS) produce a protective effect against diabetic-induced neurodegeneration in the retina of male db/db mice by preserving the functions and histology of various retinal neural cell types. The retina of db/db mice in the treatment group also exhibited an alleviated level of MMP-9 and stronger mitochondrial function compared to the controls. These findings provide further insight into the role of mitochondrial dysfunction in the development of diabetic-induced retinal neurodegeneration and suggest that topical application of CoQ10 eyedrops (conjugated with Vitamin E TPGS) may serve as a complementary therapy against DR-related neuropathy.

## Data availability statement

The raw data supporting the conclusions of this article will be made available by the authors, without undue reservation.

## Ethics statement

The animal study was approved by the Animal Subjects Ethics Sub-committee (ASESC) of The Hong Kong Polytechnic University. The study was conducted in accordance with the local legislation and institutional requirements.

## Author contributions

CL: Conceptualization, Formal analysis, Funding acquisition, Investigation, Methodology, Project administration, Visualization, Writing – original draft. BZ: Investigation, Writing – review & editing. HC: Resources, Supervision, Writing – review & editing. T-WL: Formal analysis, Software, Visualization, Writing – review & editing. SA: Investigation, Writing – review & editing. KC: Investigation, Writing – review & editing. DT: Conceptualization, Funding acquisition, Methodology, Resources, Supervision, Writing – review & editing.
