# Supplementary material for: Coenzyme Q10 eyedrops conjugated with vitamin E TPGS alleviate neurodegeneration and mitochondrial dysfunction in the diabetic mouse retina
Source: Front Cell Neurosci. 2024 May 28;18:1404987. doi: 10.3389/fncel.2024.1404987 (PMC11165046; doi:10.3389/fncel.2024.1404987)
Supplement: Supplementary file 1 [file Table_1.docx]

Supplementary Material

# Supplementary Table S1. The averaged total food and water consumption of the control db/db mice and CoQ10 treated db/db mice over the experimental period.

|  |  | Mean ± SEM (g) | p-value* |
| --- | --- | --- | --- |
| Total food consumption | db/db control | 809.83±99.93 | 0.582 |
|  | CoQ10 treated | 776.06±41.53 |  |
| Total water consumption | db/db control | 2167.83±154.39 | 0.587 |
|  | CoQ10 treated | 2054.68±131.99 |  |

*: Independent sample t-test.

## Supplementary Table S2. List of primary and secondary antibodies used in IHC.

| Primary antibodies | | | |
| --- | --- | --- | --- |
|  | Supplier | Ref # | dilution |
| *GNAT2* | LifeSpan BioScience | LS-C321680 | 1:75 |
| *PKCα* | Santa Cruz Biotechnology | sc-8393 | 1:50 |
| *Bassoon* | Cell Signaling Technology | D63B6 | 1:200 |
| *MMP-9* | Invitrogen | MA5-13595 | 1:100 |
| *GFAP* | Merckmilipore | AB5541 | 1:500 |
| *iba-1* | abcam | Ab5076 | 1:500 |
| Secondary antibodies | | | |
| Conjugate | Supplier | Ref # | dilution |
| *Cy3* | Millipore | AP192C | 1:200 |
| *Alexa Fluor 488* | Invitrogen | A21206 | 1:1000 |
|  |  | A32814 | 1:1000 |
| *Alexa Fluor 594* | Invitrogen | A21203 | 1:500 |
| *Alexa Fluor 647* | Invitrogen | A31573 | 1:1000 |

## Supplementary Figures


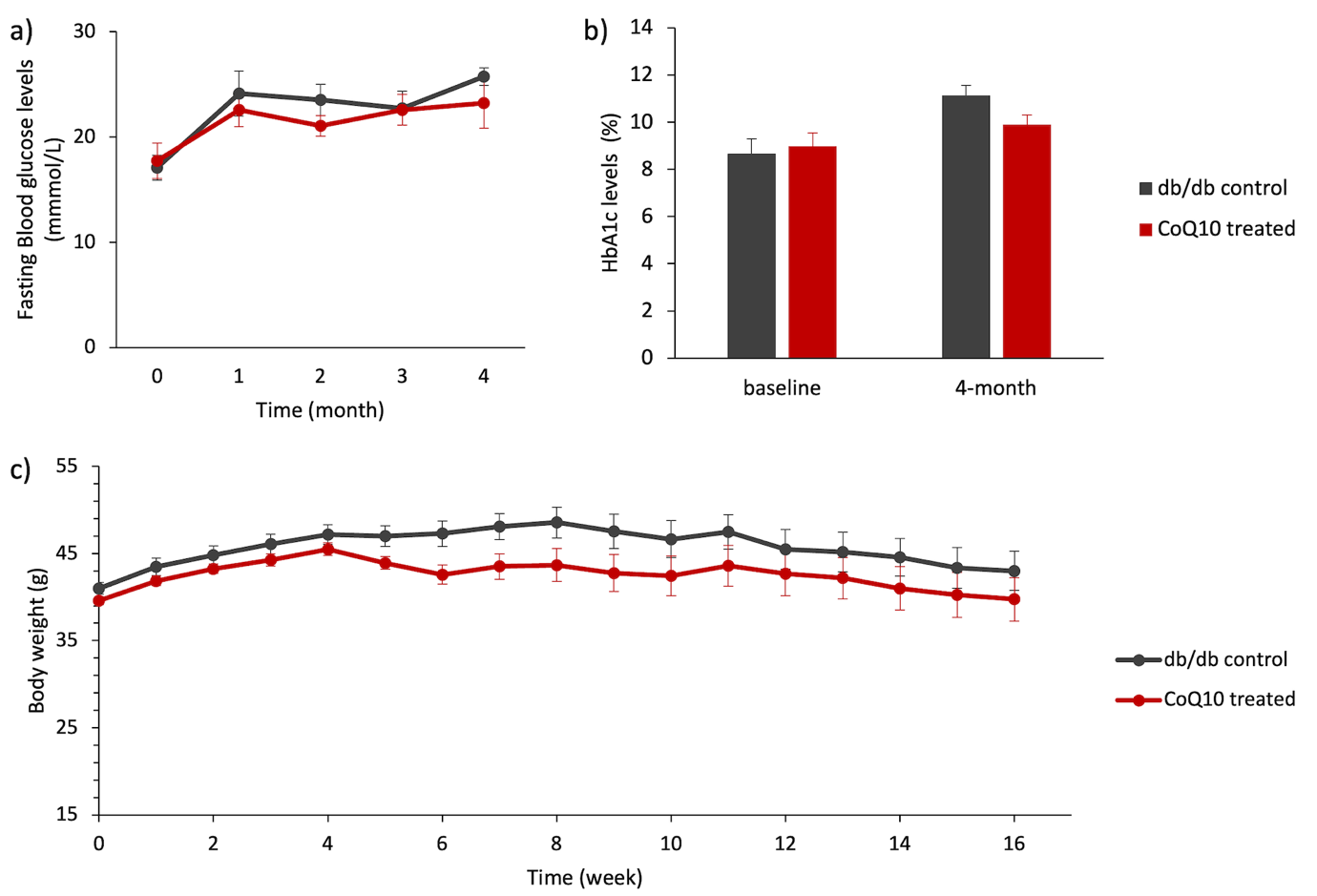


**Supplementary Figure 1.** The (a) Fasting blood glucose , (b) HbA1c levels, and (c) body weight of control db/db mice (n=11) and CoQ10 treated mice (n=12) over the experimental period (Data presented as means ± SEM.).


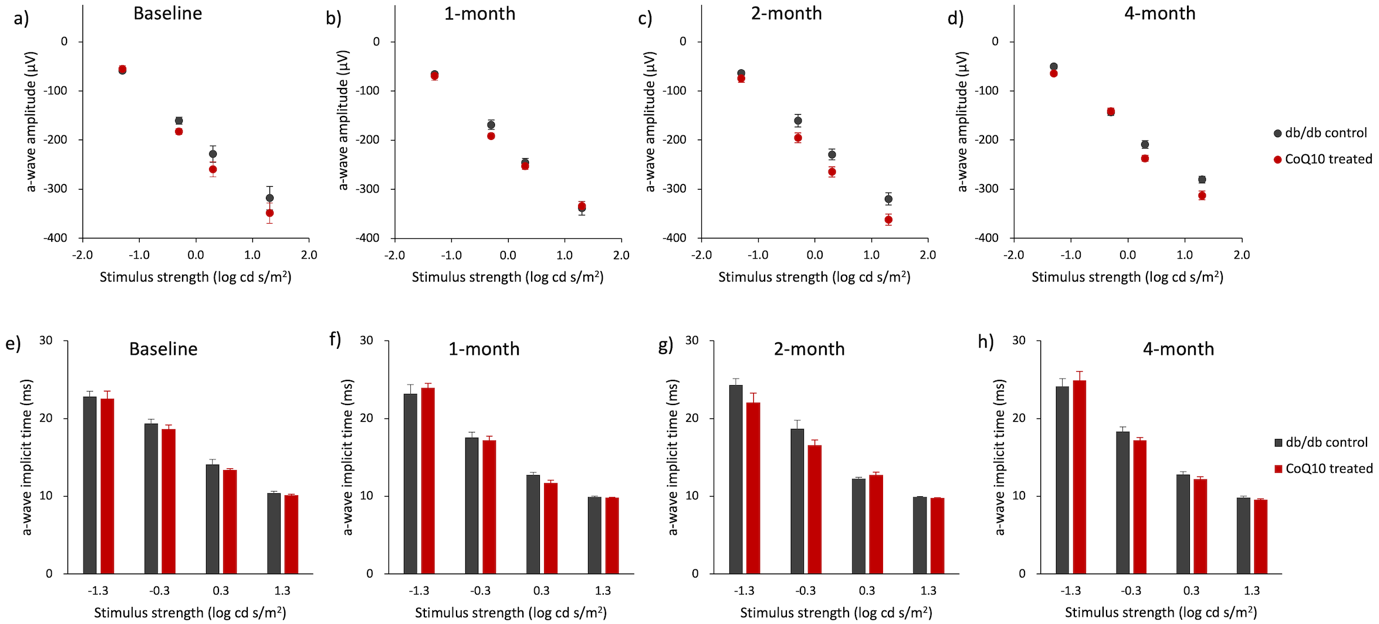


**Supplementary Figure 2.** The scotopic ERG a-wave amplitude (a-d) and implicit time (e-h) of the control db/db mice (n=11) and CoQ10 treated mice (n=12) over the experimental period (Data presented as means ± SEM.).


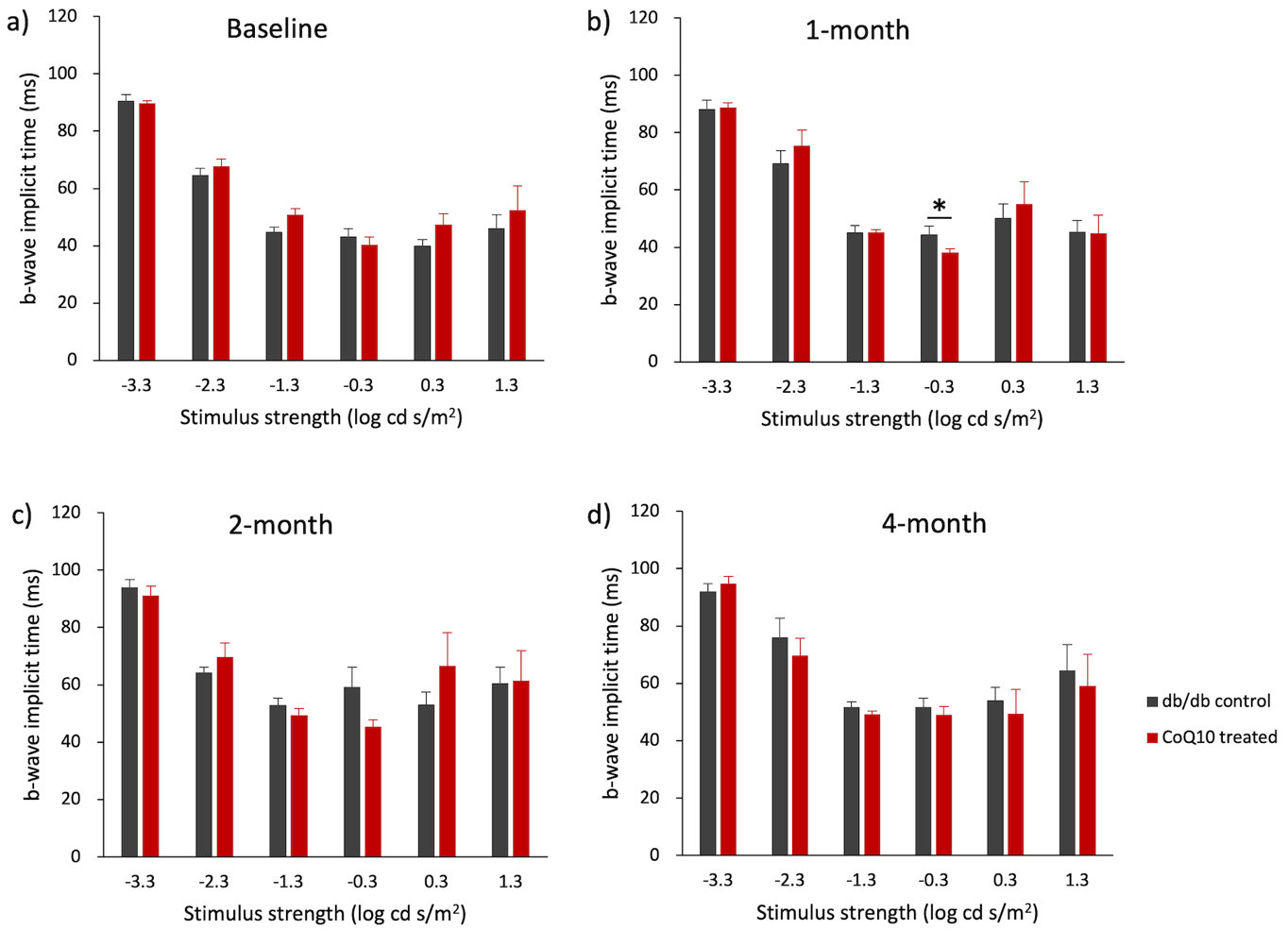


**Supplementary Figure 3.** The Scotopic ERG b-wave implicit time of control db/db mice (n=11) and CoQ10 treated mice (n=12) over the experimental period (Data presented as means ± SEM. Simple main effect analysis: * p<0.05.).


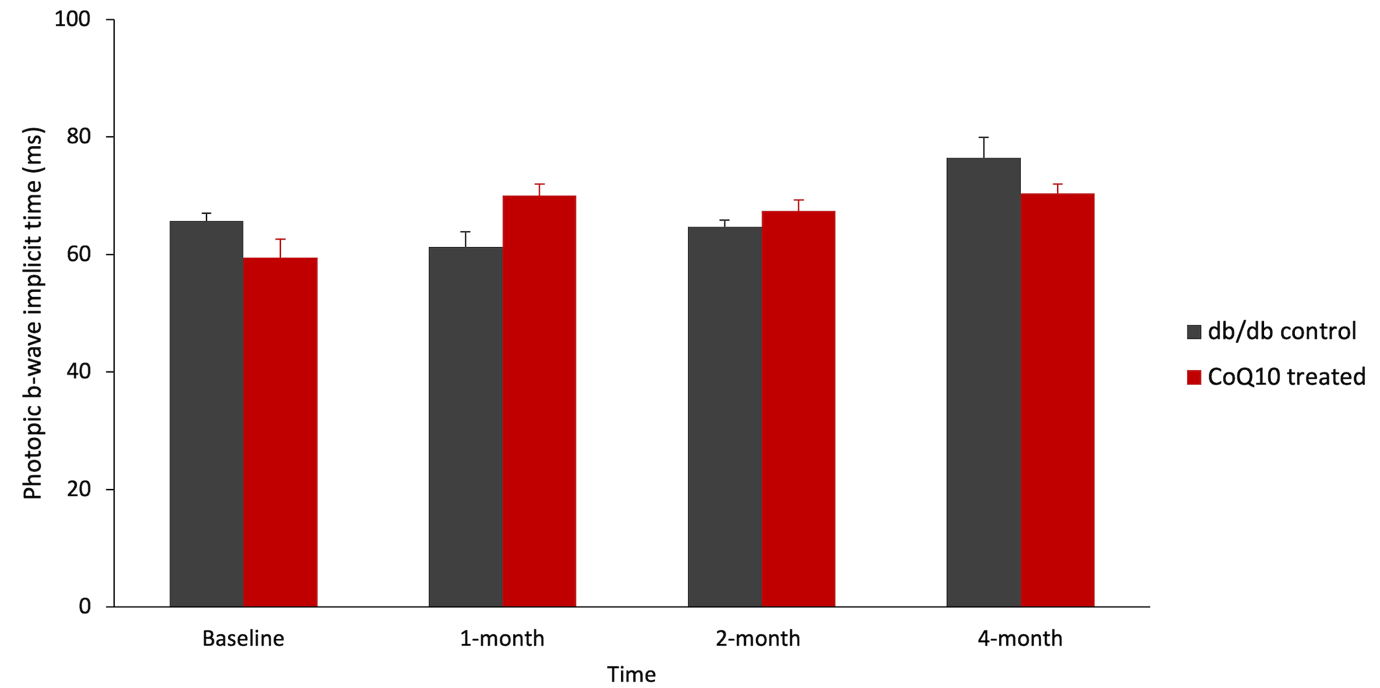


**Supplementary Figure 4.** The Photopic ERG b-wave implicit time measured in a subgroup of control db/db mice (n=5) and CoQ10 treated mice (n=5) at +0.47 log cd s/m^2^ over the experimental period (Data presented as means ± SEM.).


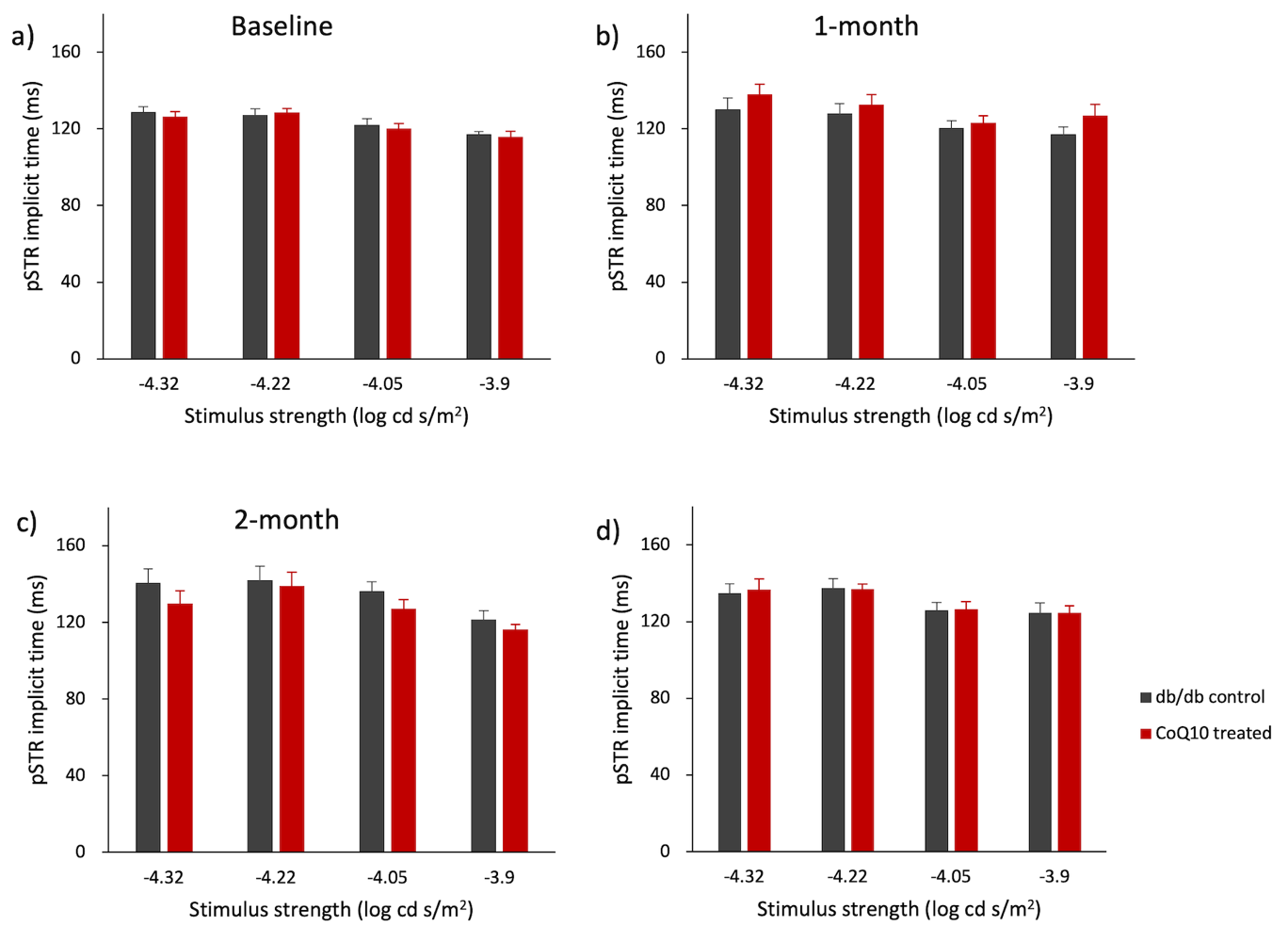


**Supplementary Figure 5.** The implicit time of positive scotopic threshold response (pSTR) of the control db/db mice (n=11) and CoQ10 treated mice (n=12) at different experimental timepoints (Data presented as means ± SEM.).


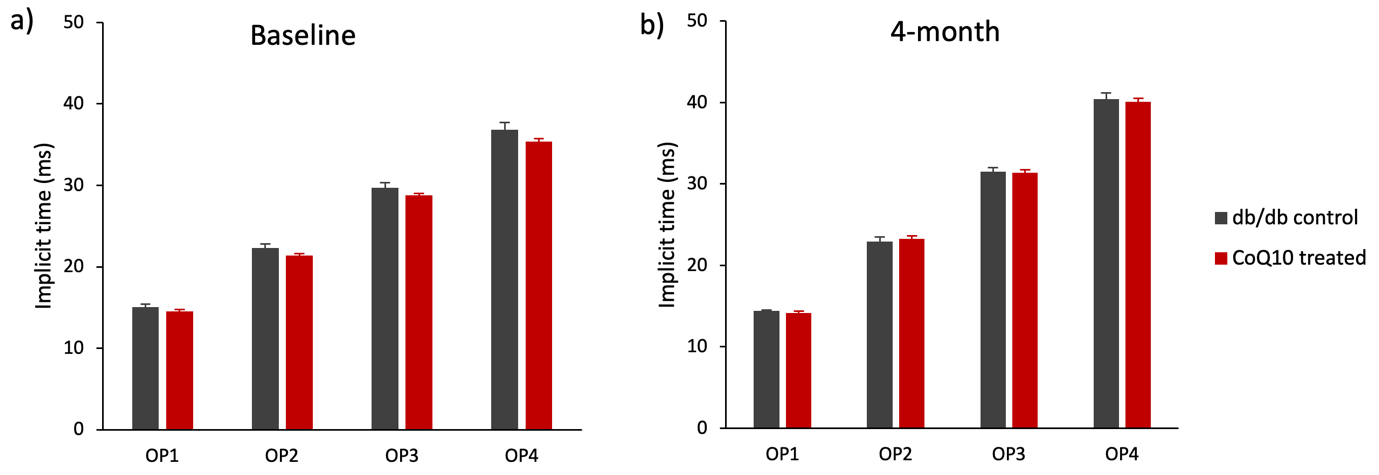


**Supplementary Figure 6.** The implicit time of the four major oscillatory potentials (OP1 – OP4) wavelets of control db/db mice (n=11) and CoQ10 treated mice (n=12) at baseline and 4-month (Data presented as means ± SEM.).
